# The influence of primary treatment approach on outcomes in patients with osteochondral fracture after patellar dislocation: a case series

**DOI:** 10.1186/s43019-023-00186-2

**Published:** 2023-04-13

**Authors:** Mikko Uimonen, Ville Ponkilainen, Ville M. Mattila, Heikki Nurmi, Juha Paloneva, Jussi P. Repo

**Affiliations:** 1 Department of Surgery, Central Finland Hospital Nova, Hoitajantie 3, 40620 Jyvaskyla, Finland; 2 Faculty of Medicine and Health Technology, Tampere University, Tampere, Finland; 3 Department of Orthopaedics and Traumatology, Unit of Muskuloskeletal Surgery, Tampere University Hospital and University of Tampere, Tampere, Finland; 4 COXA Hospital for Joint Replacement, Tampere, Finland; 5 University of Eastern Finland, Kuopio, Finland

## Abstract

**Background:**

We characterized the relation of primary treatment approaches to the need of later surgical interventions and the outcomes of patellar dislocation in patients with patellofemoral osteochondral fracture (OCF).

**Methods:**

Overall, 134 patients with OCF were categorized in two groups according to treatment approach: primary surgery (operation within 90 days from injury) and conservative treatment. Data on surgical procedures, OCF characteristics, and patellofemoral anatomy were retrospectively collected. To measure subjective outcomes, 54 patients completed the knee-specific patient-reported outcome measures (PROMs) Kujala score, Tegner activity scale, the knee injury and osteoarthritis outcome score (KOOS) quality of life (QoL) subscale, and visual analog scale pain items.

**Results:**

The mean follow-up time was 4.9 years [standard deviation (SD) 2.7 years]. The primary treatment approach was surgery in 73 patients (54%) and conservative in 61 patients (46%) of whim 18 (30%) needed late surgery. Of primary surgery patients, the OCF was reimplanted in 45 patients (62%) and removed in the rest. Of all patients, 31 needed surgery in the later phase after the primary treatment approach (either reoperation or surgery after insufficient outcome of conservative treatment). In conservatively treated patients, OCF was smaller and patellofemoral joint malformation was more severe than in surgery group. Among patients who completed the PROMs, the outcomes appeared generally acceptable in both groups.

**Conclusions:**

Although a majority of the primary treatment approaches for OCF after patellar dislocation were definitive, one-fourth of patients required surgery in the later phase. PROMs did not indicate major differences between the study groups.

## Introduction

Osteochondral fractures (OCFs) of the patellofemoral joint are common concomitant injuries after patellar dislocation [1]. OCF has been reported to occur in 38–39% of patellar dislocation patients [2, 3]. However, controversies still exist regarding the best treatment for these patients [4]. Although primary patellar dislocations are usually treated conservatively, the occurrence of OCF has been regarded in selected cases as a justification for surgery after primary dislocation [4, 5].

During past decades, surgical and arthroscopic techniques for the reimplantation of the OCF have advanced remarkably [6–10]. Furthermore, other techniques to restore the articular cartilage, such as mosaicplasty and autologous chondrocyte implantation, have been developed [11–13]. In addition, surgical patellar-stabilizing techniques, such as medial patellofemoral ligament (MPFL) reconstruction, have also been progressively developed [10, 14–20].

Since large chondral lesions have been shown to accelerate cartilaginous degeneration [21, 22], appropriate treatment for OCF is essential to secure the patellofemoral articular cartilage in the long term. Furthermore, since patellar instability may persist after primary dislocation [23], patients with OCF encounter two problems: recurrent instability and accelerated cartilaginous injury-related degeneration. Thus, planning the most appropriate treatment for these patients may be challenging since there is a scarcity of research on these patients.

In this study, we aimed to characterize the treatment approaches, primary surgery or conservative treatment, and their relation to the need for later surgical interventions and the outcomes of patellar dislocation in patients with patellofemoral OCF. The hypothesis was that, due to complexity of the patellofemoral problematics of OCF patients, the rate of later interventions would be high.

## Materials and methods

### Patient sample

**Fig. 1:**
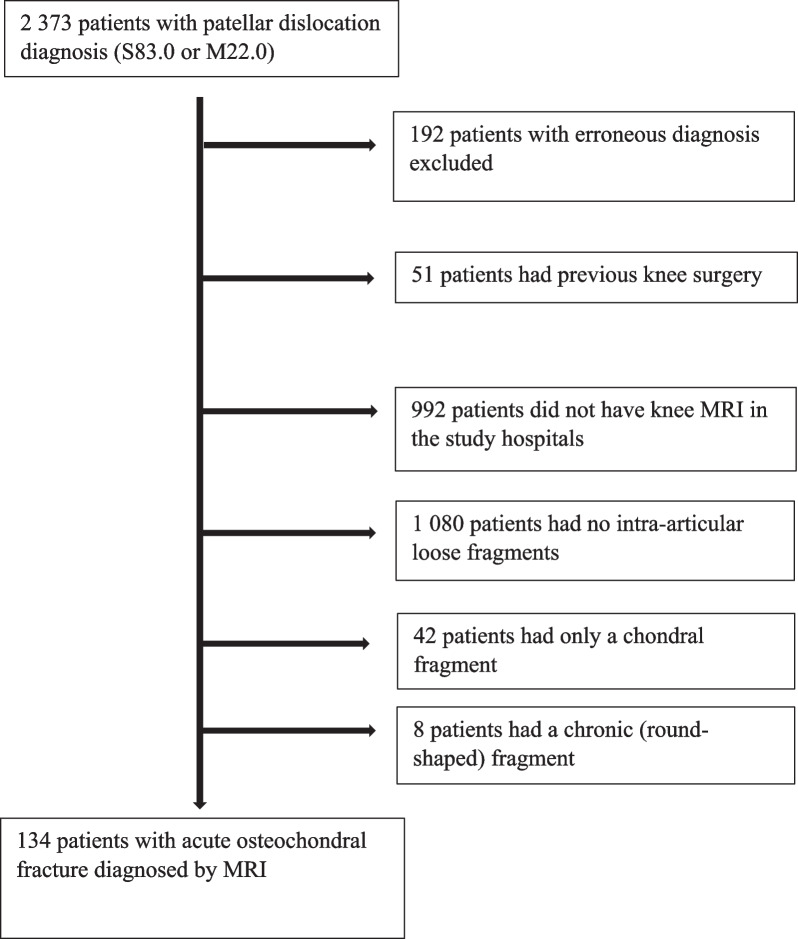
Flowschart of patient selection

This study was conducted as a multicenter study in two large hospitals in Finland (Hospital Nova of Central Finland, Jyväskylä; Tampere University Hospital, Tampere) covering a catchment population of 800,000 inhabitants. Due to a register-based study design that did not influence the treatment of the patients, ethical committee approval was not required. The research committees of the participating hospitals gave permission to conduct the study (permit ID: R19529). The study sample consisted of all patellar dislocation patients with concomitant OCF treated in the study hospitals between 2012 and 2018. Eligible patients were identified from electronic medical records using the International Classification of Diseases, tenth revision (ICD-10) diagnosis codes S83.0 (*acute patellar dislocation*) and M22.0 (*recurrent patellar dislocation*). The inclusion criterion was a diagnosis of patellar dislocation and patellofemoral OCF verified using magnetic resonance imaging (MRI). The definition for OCF was an intra-articular loose-body-containing components of both cartilage and bone. The search identified a total of 134 eligible patients. A flowchart describing patient selection is presented in Fig. 1.

Data on patient and clinical characteristics were collected retrospectively from electronic patient records. The data consisted of information on previous patellar dislocations before the expected occurrence of OCF, primary treatment approach (conservative or surgery), late surgery (i.e., performing of surgical procedures related to patellar dislocation after the first 90 days from expected occurrence of OCF), reoperations (performing of later surgical operations for patellar dislocation or osteochondral fracture), and later recurrent patellar dislocations (i.e., recurrent patellar dislocation after the primary treatment decision). In surgical operations, management of OCF (reimplantation versus removal) as well as the performing of other cartilage restoration chondroplasties or patellar-stabilizing procedures were examined. The follow-up time was calculated from the expected occurrence of OCF (i.e., time of patellar dislocation after which the OCF was detected in MRI) to the end of 2018 or the date on which the outcome questionnaires were administered (minimum follow-up, 6.5 months).

### Treatment protocol

**Fig. 2:**
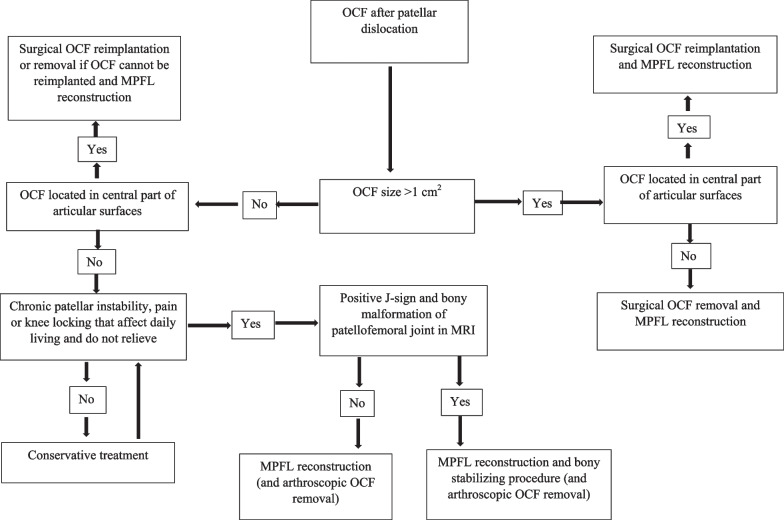
Pragmatic treatment protocol

The principles of the study protocol are presented in Fig. 2. In the study hospitals, the treatment decision in the primary phase after occurrence of radiologically demonstrated OCF after patellar dislocation was based on OCF size and location as well as history of patellar instability. OCF sized 1 cm^2^ or more in the central part of the articular surface was considered to be a relative indication for surgery in the primary phase. In all surgeries, knee arthroscopy was conducted routinely to inspect the condition of cartilage surfaces and any cartilage defects. Our primary aim was to reimplant the OCF into its origin using absorbable pins, nails, screws, or sutures. If the reimplantation failed or was not possible, the OCF was removed. Other cartilage restoration chondroplasties, such as microfractures, mosaicplasty, and osteochondral grafting, were not performed in the primary surgery. In addition, MPFL reconstruction was routinely performed in all patients, with a few exceptions, at the beginning of the study period. Bony stabilizing procedures, such as trochleoplasty, tibial tubercle osteotomy, or femoral osteotomy, were not performed in the primary phase.

In patients with small OCF (< 1 cm^2^) located in the outer edge of the patellofemoral surface, conservative treatment was the primary choice. Surgery was considered if the patient had previous patellar dislocations or obvious clinically verified patellar instability. In these cases, MPFL reconstruction was the primary procedure, and it was performed in practically every patient. Additional bony stabilizing procedures were performed in case of positive J-sign in clinical examination and apparent bony deformity in the knee MRI. The type of bony procedure, trochleoplasty, tibial tubercle or femoral osteotomy, or a combination of these, was selected with respect to the patellofemoral anatomy of the patient and the preferences of the operating surgeon. If the OCF size was 1 cm^2^ or more and located in the outer edge of the patellofemoral joint, it was removed arthroscopically without cartilage restoration procedures.

In the later phase after undergoing the primary treatment, either conservative or surgery, the patient might have sought further treatment if symptoms, such as pain, knee locking, or chronic patellar instability, of the injured knee were not relieved and affected daily living. Knee pain related to chondral injury, an intra-articular loose fragment, or persistent patellar instability was an indication for later-phase surgery. In later-phase surgery, a possibly symptomatic loose OCF was removed arthroscopically. In addition, arthroscopic debridement of the patellofemoral cartilage surfaces was performed. In patients with an unrecovered chondral lesion in the central part of articular surface, cartilage restoration chondroplasty was used. The management of persistent patellar instability was the same in both the primary and later phases and was performed according to patient anatomy.

The post-operative rehabilitation protocol was similar in all surgeries. Full weight-bearing on a straight leg was allowed immediately after the operation. Stair-walking was to be avoided for 6 weeks. Riding a bicycle and minor squat exercises began at 6 weeks. Closed kinetic chain exercises (hack squat and leg press) with progressively increasing force were recommended from 6 weeks to 2 months. Squat exercises with light additional weights and jogging were recommended after 3 months. Unlimited exercise and movements were allowed from 4 to 6 months.

### Radiologic examination

The MRIs of the injured knees were examined for patellofemoral anatomy and OCF characteristics. The MRI scans were conducted using a 1.5T or 3T magnet strength scanner with a coil. Images were structured using proton density and T2-weighted turbo spin echo sequences with a slice thickness of between 2.5 mm and 3.5 mm and slice increment of between 2.8 mm and 4.0 mm. During the scan, the knee was set in 20–30° of flexion. In addition, OCF location (patella versus femur) and size were examined in patients in the OCF group.

Anatomical parameters related to patellar instability were measured from the MRIs. Measurements for each patient were performed case-by-case by two independent observers from the two study hospitals, and the final measurement was calculated as the mean of the measurements of the two observers. The Insall–Salvati index (ISI) [24], Caton–Deschamps index (CDI) [25], and patellotrochlear index (PTI) [26] were measured to assess the patellar height. The lateralization of patellar tracking was examined by measuring the tibial tubercle–trochlear groove (TT-TG) distance [27] and the tibial tubercle–posterior cruciate ligament distance (TT-PCL) [28]. Trochlear configuration was investigated by measuring the trochlear sulcus angle [29], trochlear depth [29], trochlear facet asymmetry ratio [29], trochlear condyle asymmetry ratio [29], and lateral trochlear inclination angle [30]. Finally, skeletal maturity was examined using a definition of an opening in the growth plates of < 5 mm in any section or the complete epiphyseal fusion of the distal femur [31]. Measurement reliability between the two observers was good in each parameter, as the calculated intraclass correlation coefficients varied between 0.76 and 0.92, with the lowest value in the sulcus angle. Values over 0.75 indicate good reliability [32].

### Treatment approach

The patients were divided into two groups according to the treatment approach: primary surgery and conservative treatment. The definition for the primary surgery group was that the patients underwent surgical procedure during the first 90 days after the expected occurrence of OCF in patellar dislocation (i.e., the patellar dislocation after which OCF was detected in knee MRI). The conservative treatment group consisted of patients that did not undergo surgical treatment for patellar dislocation or OCF within 90 days from injury. The main outcomes examined were whether the primary treatment approach was definitive (i.e., whether there were later surgical procedures after the primary treatment) and whether there were recurrent patellar dislocations after the primary treatment decision.

### Subjective outcomes

To investigate subjective outcomes of the patients, patient-reported outcome measures [PROMs; Kujala score, KOOS quality of life subscale, Tegner activity scale, and visual analog scale (VAS) pain items] were sent to patients via mail along with a prepaid return envelope. In addition, patients were asked to complete an item on overall satisfaction with the knee condition on a 0–100 scale, with a higher score indicating higher satisfaction.

### Statistical analysis

The results are presented as counts and percentages or means and standard deviations (SD). Comparisons of continuous variables were performed using an independent sample *t*-test, whereas categorical variables were compared using a chi-square test. All analyses were conducted using R 4.0.3 software.

## Results

The mean age of the patients was 22.0 years (SD 8.4 years), and a slight majority was female (*n* = 74, 55%). In 88 patients (66%), the physes were closed. The mean follow-up time was 4.9 years (SD 2.7 years). The patients in the surgery group were younger than the patients in the conservative group (*p* = 0.020, Table 1).

**Table 1:** Patient characteristics

	Surgery, *n* = 73	Conservative, *n* = 61	*p*-Value*
Female, *n* (%)	40 (55)	22 (56)	1.000
Age during the occurrence of OCF, mean (SD)	20.4 (7.4)	23.8 (9.2)	0.020
Closed physes, *n* (%)	43 (59)	45 (74)	0.105
BMI, mean (SD)	27.9 (5.3)	28.0 (8.9)	0.971
Follow-up duration in years, mean (SD)	4.6 (2.5)	5.3 (3.0)	0.203

*OCF* osteochondral fracture, *SD* standard deviation, *BMI* body mass index

*Calculated using an independent sample t-test in continuous variables or chi-square test in categorical variables

**Fig. 3:**
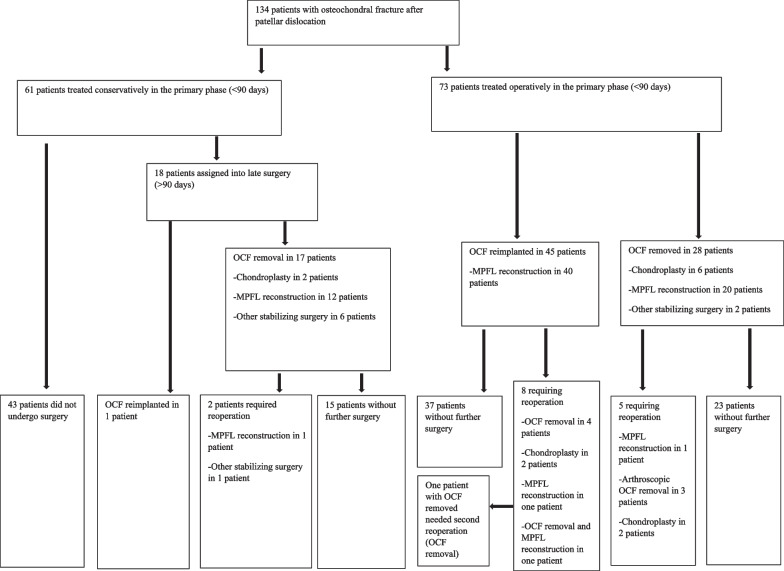
Flowchart of treatment approaches

Of the 134 patients, the primary treatment approach was conservative in 61 patients (46%) and surgery in 73 patients (54%; Fig. 3). Eighteen patients (30%) who were treated conservatively were assigned to late surgery (> 90 days after the occurrence of OCF). On 12 of these patients, MPFL reconstruction or other stabilizing surgery was performed due to persistent patellar instability. In the rest, the indication for surgery was pain, crepitation, or locking of the injured knee. In 17 out of 18 patients assigned to late surgery, OCF was removed. In two patients with the OCF removed, chondroplasty was performed. After late surgery, three patients needed reoperation; two patients with the OCF removed needed subsequent stabilizing surgery, and one patient with the OCF reimplanted in the first surgery needed arthroscopic removal of another intra-articular loose fragment.

Of the 73 patients treated surgically in the primary phase, the OCF was reimplanted in 45 (62%) patients and removed in the rest (Fig. 3). MPFL reconstruction was performed in 60 patients (82%). In two of these patients (3%), another stabilizing procedure was performed simultaneously. Furthermore, eight patients (18%) with primarily reimplanted an OCF required reoperation. Of those patients who had an OCF removed, five (18%) required reoperations (OCF was removed in two patients, chondroplasty was performed in two patients, and OCF removal and MPFL reconstruction were carried out in one patient).

The proportion of patients with OCF after primary patellar dislocation was lower in the conservative than in surgery group (*p* = 0.026, Table 2). In the majority of patients, the OCF was in the medial patellar facet (46%) or in the lateral femoral condyle (34%). The OCF size was larger in surgery patients (*p* < 0.001). Of patellofemoral anatomy parameters, PTI showed more severe patella alta in the conservative group than in surgery group (*p* = 0.020). In addition, trochlear condyle asymmetry showed a tendency toward a more prominent lateral condyle in the surgery group than in conservatively treated patients (*p* = 0.058).

**Table 2:** Clinical characteristics in the treatment groups

	Surgery, *n* = 73	Conservative, *n* = 61	*p*-value*
OCF occurred in primary dislocation, *n* (%)	63 (86)	42 (69)	0.026
OCF location, *n* (%)			0.351
Patellar medial facet	30 (41)	32 (52)	
Patellar ridge	15 (21)	7 (11)	
Femur lateral condyle	25 (34)	21 (34)	
Femur and patella	3 (4)	1 (2)	
OCF size in cm^2^, mean (SD)	2.6 (1.7)	1.4 (0.9)	< 0.001
Anatomical measures, mean (SD)			
ISI	1.18 (0.18)	1.22 (0.21)	0.322
CDI	1.17 (0.14)	1.20 (0.17)	0.335
PTI	0.56 (0.11)	0.51 (0.14)	0.020
TT-TG	14.8 (4.4)	13.7 (4.3)	0.163
TT-PCL	21.8 (3.7)	21.4 (3.4)	0.593
Sulcus angle	154.9 (7.1)	154.4 (7.6)	0.718
Trochlear depth	2.5 (1.0)	2.6 (1.1)	0.425
Trochlear facet asymmetry	0.51 (0.17)	0.56 (0.13)	0.127
Trochlear condyle asymmetry	1.044 (0.026)	1.035 (0.029)	0.058
Lateral trochlear inclination angle	13.3 (4.1)	14.1 (4.8)	0.318
Recurrent patellar dislocations after occurrence of OCF, *n* (%)	10 (14)	11 (18)	0.654

*OCF* osteochondral fracture, *SD* standard deviation, *ISI* Insall–Salvati index, *CDI* Caton–Deschamps index, *PTI* patellotrochlear index, *TT-TG* tibial tubercle–trochlear groove distance, *TT-PCL* tibial tubercle–posterior cruciate ligament distance

*Calculated using an independent sample *t*-test in continuous variables or Chi-square test in categorical variables

Out of 134 patients, 54 returned completed questionnaires, resulting in response rate of 40%. The patient-reported outcomes showed acceptable outcomes for all patients, although KOOS QoL scores indicated knee-related impairments in the quality of life of the patients in both groups (Table 3). There were no prominent differences between the groups.

**Table 3:** Demographic characteristics and responses of the patients in the treatment groups who returned completed patient-reported outcome measure questionnaires

	Surgery	Conservative	*p*-Value*
Response rate, *n* (%)	30 (41)	24 (39)	
Female, *n* (%)	14 (47)	8 (47)	1.000
Age, mean (SD)	25.7 (7.5)	30.4 (11.9)	0.114
Recurrent patellar dislocations after occurrence of OCF, *n* (%)	5 (17)	5 (21)	0.969
PROM scores, mean (SD)			
Kujala score	85.3 (11.3)	86.3 (14.7)	0.778
Tegner score	5.2 (1.7)	5.9 (1.9)	0.181
KOOS QoL	70.0 (20.5)	70.6 (21.3)	0.921
VAS pain, mm			
Overall	16.1 (17.8)	14.8 (22.5)	0.820
Day	8.5 (12.8)	9.0 (18.7)	0.894
Night	3.5 (5.6)	5.0 (12.9)	0.560
Movement	18.7 (21.1)	16.9 (25.3)	0.773
Rest	4.3 (9.4)	5.7 (11.8)	0.619
Overall knee condition	84.9 (12.5)	81.9 (16.5)	0.451

*OCF* osteochondral fracture, *SD* standard deviation, *PROM* patient reported outcome measure

*Calculated using an independent sample *t*-test in continuous variables or chi-square test in categorical variables

## Discussion

In this study, the primary treatment approach, either conservative or surgery, was definitive in the majority of patients. However, one-fourth of patients needed later surgery. In addition, a relatively high proportion of patients who had undergone surgery needed a reoperation. Patient-reported outcomes were generally good in most of the patients.

Based on our experience, in the primary phase after patellar dislocation with concomitant OCF, the treatment decision approach should be based on the clinical characteristics of the patient and the injury. These characteristics include the location and size of the OCF, history of chronic patellar instability, anatomical and clinical findings related to patellar instability, and the individual needs of the patient. Surgery in the primary phase is targeted at restoring the articular cartilage and stabilizing the patella to prevent chronic instability. In the later phase, the importance of the patient’s symptoms in making decisions on possible surgical procedures is emphasized, and the procedures may be more targeted at treating particular symptoms. This philosophy was reflected in the treatment protocol of the patients in the present study.

As expected, the size of OCF was largest in patients treated surgically in the primary phase. In the majority of patients who underwent primary surgery, the OCF was reimplanted. Further, it is also likely that a proportion of the removed OCFs were removed after unsuccessful reimplantation. A larger OCF size in the primary surgery group suggests a higher willingness to attempt reimplantation of OCF primarily. A smaller OCF size in patients treated conservatively may indicate a less severe lesion in the articular cartilage as well as later symptoms. Of the patients that were treated conservatively in the primary phase but needed later surgery, reimplantation was performed on one patient. These findings are in line with the main goal of salvaging and restoring the articular cartilage in primary surgery, as chondral lesions with a diameter of over 10 mm have been shown to accelerate cartilaginous degeneration [21, 22]. In later surgery, restoring of the cartilage was no longer the primary target. Late surgery was targeted to manage symptoms caused by a floating loose fragment, such as knee locking, crepitation, and pain, as well as persistent patellar instability. According to our findings, in five out of 45 patients (11%) with the OCF reimplanted, the implanted OCF failed to attach sufficiently, and later removal of the fragment was needed. A high proportion of patients in the surgery group had undergone MPFL reconstruction together with the removal or fixation of the OCF fragment. This reflects the trend toward preventive procedures against recurrent patellar dislocation even in cases where the OCF occurred in the first patellar dislocation. Cartilage restoration techniques other than reimplantation of the OCF, such as microfracturing, osteochondral autografts, and autologous chondrocyte implantation, were only used on rare occasions.

Patellofemoral anatomy and OCF location differed only modestly between the groups, although the number of patients may lack power to detect differences. In the conservatively treated patients, lower PTI indicated a more pronounced patellar height than in the surgery group, whereas higher trochlear condyle asymmetry indicated more prominent lateral femoral condyle in the surgically treated patients. Differences in the other anatomical measures were modest. Furthermore, the proportion of patients who had had previous patellar dislocations before the occurrence of OCF was higher in the conservatively treated patients. The trauma energy required to dislocate the patella may be higher in patients with more normal anatomy and without previous dislocations leading to larger OCF [39]. Further, the groups differed with regards to OCF size, which was larger in surgery patients. These findings are in line with the assumption that trauma energy required to dislocate the patella may be higher in patients with more normal anatomy and without previous dislocations leading to larger OCF and further to early surgical treatment [39]. Indeed, OCF size plays a key role in determining the primary treatment approach. In the later phase, those patients presenting with persistent instability or other symptoms related to an intra-articular loose fragment are referred to late surgery, while the others are treated according to the primary treatment decision. After follow-up, the patients in both groups reported relatively good subjective outcomes regarding functionality, whereas in pain- and quality-of-life-related outcomes, slight impairments were observed.

In summary, the treatment of patellar dislocation with concomitant OCF is challenging owing to two-dimensional nature of the problem, namely, articular cartilage lesion and patellar instability. According to this study, the outcomes achieved by the presented protocol are generally acceptable, although surgical procedures, either late surgery or reoperations, were occasionally needed after the primary treatment decision. As the outcomes were comparable between primary surgery patients with OCF either reimplanted or removed, it seems likely that primary reimplantation may not be necessary for all patients. Nevertheless, further research is needed to provide insights into which patients could be treated conservatively in the primary phase and which would benefit more from primary OCF reimplantation. Furthermore, the differences in the long-term outcomes of patients with OCF reimplanted and removed are still poorly understood.

The main limitation of the current case series was the retrospective study design that encompasses specific shortcomings. The occurrence date of the OCF was not certain, as the date was determined according to the MRI during which the OCF was firstly detected. This may have led to possible inaccuracies regarding the occurrence dates. Moreover, it is possible that a small proportion of patients may have sought help from private hospitals rather than from the study hospitals. In these patients, some of the late surgeries may have been missed. The number of patients that completed the outcome measure questionnaires was small, resulting in heterogeneity between groups. Furthermore, we could not retrieve information on other potential factors related to patellar instability and OCF risk such as trauma mechanism and generalized joint laxity. In addition, due to the non-randomized study setting without standardized protocols for conservative treatment or rehabilitation, we could not account for the actual effect of conservative treatment. However, we addressed a representative sample of patients with patellar dislocation and concomitant OCF from two large hospitals. Data for each individual patient were extensive and granular.

## Conclusion

Although a majority of the primary treatment approaches for OCF after patellar dislocation were definitive, one-fourth of patients required surgery in the later phase. PROMs did not indicate major differences between study groups.

## Data Availability

Data generated and analyzed during this research are available from the corresponding author on reasonable request.
